# Experiment on the Properties of Soda Residue-Activated Ground Granulated Blast Furnace Slag Mortars with Different Activators

**DOI:** 10.3390/ma15103578

**Published:** 2022-05-17

**Authors:** Yonghui Lin, Dongqiang Xu, Wenguang Ji, Xianhui Zhao

**Affiliations:** 1 School of Civil and Transportation Engineering, Hebei University of Technology, Tianjin 300401, China; 2002021@hebut.edu.cn; 2 Department of Economics and Management, Hebei Normal University for Nationalities, Chengde 067000, China; zhxyjwg@163.com; 3 School of Civil Engineering, Hebei University of Engineering, Handan 056038, China; zhaoxianhui@hebeu.edu.cn

**Keywords:** soda residue, ground granulated blast furnace slag, activator, mortar, orthogonal experiment, mechanical strength, hydration products, microstructure

## Abstract

Soda residue (SR), a solid waste generated in the production of Na_2_CO_3_ during the ammonia soda process, with a high pH value of 12, can be used as an activator of alkali-activated ground granulated blast furnace slag (GGBFS) cementitious materials. Three groups of experiments on SR-activated GGBFS mortars were designed in this paper to assess the role of the dominant parameters on fluidity and compressive strength of mortars. The results indicate that for fluidity and mechanical properties, the optimal scheme of SR-activated GGBFS mortars is 16:84–24:76 S/G, 0.01 NaOH/b, 0.05 CaO/b, and 0.50 w/b, with fluidity and compressive strength (28 d) of the mortars being 181–195 mm and 32.3–35.4 MPa, respectively. Between 2.5–10% CaCl_2_ addition to CaO (5%)-SR (24%)-activated GGBFS mortar is beneficial to the improvement of the compressive strength of C2, whereas the addition of CaSO_4_ is harmful. The main hydration products of mortars are ettringite, Friedel’s slat, and CSH gels. The results provide a theoretical basis and data support for the utilization of SR.

## 1. Introduction

Soda residue (SR) is an industrial solid waste of soda ash production by an ammonia-alkali process [1]. From 2017 to 2020, about 13 million tons of soda ash were produced annually through an ammonia-alkali process in China [2]. The production of 1 ton of soda ash generates about 0.3–0.6 tons of SR [3]. Thus, at least 3.9 million tons of SR are produced each year. However, only 5% of SR is recycled, mainly used to fill roadbeds [4]. Large amounts of untreated SR not only occupy land (In China, more than 15 km^2^ of land was occupied), polluting the environment [4], but also lead to a waste of resources, so how to recycle it has become an urgent problem to be solved. Cement and alkali-activated slag cementitious materials (AAS) are one of the most widely used building materials at present. The cement-based cementitious materials made from SR could be an effective way to utilize SR in large quantities.

Ground granulated blast furnace slag (GGBFS) is a by-product material of pig iron [5,6]. Chemical components of GGBFS are mainly 27–40% SiO_2_, 30–50% CaO, 5–15% Al_2_O_3_, and 1–10% MgO [5]. GGBFS can be activated by alkaline materials (such as NaOH and CaO et al.) to generate AAS. Compared with Portland cement (PC), AAS can reduce carbon emissions [7], as traditional alkaline activators (NaOH, Na_2_SiO_3_) are expensive and have a great impact on the environment [8,9]. Ca(OH)_2_ and CaO are potential alternatives to alkali activators because CaO (or Ca(OH)_2_)-activated slag cementitious materials can achieve high 28 d compressive strength [10,11]. In addition, both Ca(OH)_2_ and CaO are much less expensive than NaOH and Na_2_SiO_3_. The main mineral phases in SR are 39.6% calcite, 11.2% portlandite, 6% NaCl, 13.4 CaCl_2_, and 9.8% CaSO_4_ [12]. Owing to the existence of portlandite, SR has a high pH value, ranging from 11.8 to 12.0, with different mass ratios of SR to water (1:1–1:150) [13]. Pan [14] and Ma [15] reported that in AAS when the pore solution’s pH value was greater than or equal to 10.5, Si and Al in slag were promoted to participate in the active reaction by destroying the glass chain on the surface of slag. Additionally, Song [16] considered that slag hydration reaction was activated or accelerated when the solution’s pH value was higher than 11.5. Thus, in terms of the pH value of the solution, SR can provide a hydration environment for slag.

Previous studies [17,18,19] investigated the usage of SR instead of limestone as a cement raw material or a mineral additive to produce cement. However, this method is not widely used due to the high concentration of chloride ions in SR, which may lead to corrosion of steel. Compared with PC, AAS has a stronger ability to solidify chloride ions [20,21] because slag is rich in Al-phase, which can react with NaCl or CaCl_2_ to form Friedel’s slat (Fs), as shown in Equations (1) and (2) [22,23]. Additionally, numerous studies [8,24,25,26] have demonstrated that SR can provide a hydration environment for AAS, and Ca^2+^, [SO_4_]^2−^ and Cl^−^ ions contained in SR can also participate in the hydration reaction of slag, generating ettringite (AFt) and Fs. However, there is still a risk of steel corrosion due to the presence of Cl^−^ ions in SR, so it is a better choice to utilize SR in the unreinforced concrete field [27,28]. Xu [27] used 30% SR, 45% GGBFS, 15% steel slag, and 10% desulfurization gypsum to prepare clinker-free concrete, and the 28 d compressive strength of the concrete could reach up to 38.3 MPa. Song [26] prepared mortar using SR, GGBFS, and cement (mass ratio was 2:6:2), and the compressive strength could reach 45.1 MPa. Sun [29] took 30% SR, 23% GGBFS, and 30% fly ash as main raw materials, adding 12% gypsum and 5% alkaline activator (Na_2_SiO_3_), and prepared cementitious materials, the compressive strength of which could reach 35.2 MPa at 28 d. Therefore, SR can be used as the raw material for the preparation of cementitious materials.

Ca(OH)_2_ + 2NaCl → CaCl_2_ + 2Na^+^ + 2OH^−^ (1)

3Ca(OH)_2_ + CaCl_2_ + Al_2_O_3_ + 7H_2_O → 3CaO·Al_2_O_3_·CaCl_2_·10H_2_O (2)

Different kinds and amounts of raw materials affect the compressive strength of cementing materials. According to tests and results of previous studies [12,13,30], the 8–24% mass ratio of SR replacing GGBFS gave high compressive strength (27.6–33.7 MPa) of the SR-activated GGBFS cementitious material at 28 d and low compressive strength (0.6–1.6 MPa) at early ages (3 d). Using SR and fly ash (mass ratio was 2:3) to prepare the geopolymer, Zhao [4] reached a similar conclusion. NaOH and CaO could activate GGBFS and dissolve its silicon and aluminum structure, generating hydration products [31]. In SR-activated GGBFS cementitious materials, the low content of NaOH (NaOH to GGBFS mass ratio was 1%) was beneficial to the improvement of compressive strength of the cementitious materials, while the high content of NaOH (5–10%) was not [30]. It might be because a low concentration of NaOH could provide a better hydration environment for slag. Relevant studies [32,33] show that pH value between 12.6 and 13.0 was the best environment for the hydration reaction of cement-based materials to produce hydration products. Additionally, the water-cement ratio also affected the compressive strength of cementitious materials [34,35]. It can be seen that the amount of SR, alkali activators (NaOH, CaO), and water-cement ratio all affect the compressive strength of SR-activated GGBFS cementitious materials.

The reason for strength enhancement derived from CaO for alkali-activated cementitious materials [36,37] is that CaO reacted with water to generate Ca(OH)_2_, which consumed water in the solution, further strengthened local alkalinity, and accelerated the release of silicon, aluminum, and calcium from raw material particles, thus facilitating the silica-aluminum polymerization reaction. Additionally, the heat released by CaO in water increased the local temperature of the solution and promoted the polymerization of silicon and aluminum [38]. Zhang [39] considered that a small amount of gypsum could accelerate the hydration of alkali-activated slag pastes and was beneficial to the formation of the skeleton structure of mortar. However, an excessive amount of gypsum caused large amounts of ettringite generation, leading to stress concentration in mortar and strength reduction of mortar. Yum [36,40] found that CaCl_2_ could improve the compressive strength of the pastes at different ages, increase the cumulative reaction heat within 72 h, accelerate the dissolution of GGBFS, and increase the number of reaction products.

In this paper, the strength development of SR-activated GGBFS mortars with different admixtures was investigated in three groups of experiments in order to explore the factors influencing compressive strength. For Group Ι, an orthogonal experiment was used to determine the effects of SR to GGBFS mass ratio, NaOH to binder (SR and GGBFS mixture) mass ratio, CaO to binder mass ratio, and water to binder mass ratio on physical and the mechanical properties of the mortars. For Group Ⅱ, the optimal mixing ratio of mortars was determined. For Group Ⅲ, experiments were designed to clarify the effects of calcium compounds with different anionic species on mechanical properties of the mortars. Finally, the hardened cementitious materials were characterized by X-ray diffraction (XRD) and scanning electron microscopy (SEM). The obtained results would provide a guideline and reference for the use of SR in the field of AAS cementitious materials.

## 2. Materials and Methods

### 2.1. Materials

**Figure 1:**
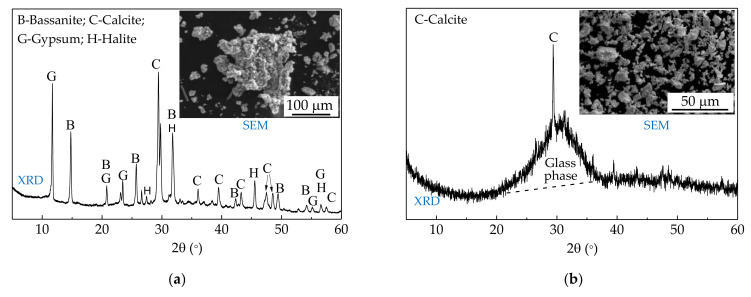
XRD patterns and SEM images: (**a**) SR; (**b**) GGBFS.

Raw materials of SR-activated GGBFS mortar are SR (provided by Tangshan Sanyou Chemical Industry Co., Ltd., Tangshan, China), GGBFS (provided by Tangshan Xinrong Slag Powder Co., Ltd., Tangshan, China), and ISO standard sand (Xiamen Iso Standard Sand Co., Ltd., Xiamen, China). NaOH, CaO, CaCl_2_, and CaSO_4_ are selected as mortar admixtures. The chemical and physical properties of SR and GGBFS are referenced in the previous studies [12,13]. The BET surface area of SR and GGBFS are 409 m^2^/kg and 450 m^2^/kg, respectively. The pH value of SR and GGBFS are 12.0 and 10.7, respectively. The XRD patterns and the SEM images of the SR and the GGBFS are shown in Figure 1.

The main mineral phases in the SR are 39.6% calcite, 11.2% portlandite, 6% NaCl, 13.4 CaCl_2_, and 9.8% CaSO_4_. The GGBFS was mainly composed of 41.4% CaO, 28.1% SiO_2_, 14.8% Al_2_O_3_, and 9.5% MgO. The specific gravity and fineness modulus of ISO standard sand are 2.6 and 2.9, respectively. All the admixtures are of analytical grade, as shown in Table 1.

### 2.2. Preparation of Specimens

Based on previous study results [12,13,30] and analysis, three groups of experiments were designed in this paper. For group Ι, an orthogonal experiment (nine kinds of mortars) was employed to assess the role of the dominant parameters with 4 factors and 3 levels, as shown in Table 2.

The 4 factors and 3 levels are: (1) SR to GGBFS mass ratio (S/G): 8:92, 16:84, and 24:76; (2) NaOH to binder (SR and GGBFS mixture) mass ratio (NaOH/b): 0, 0.005, and 0.01; (3) CaO to binder mass ratio (CaO/b): 0, 0.025, and 0.05; (4) water to binder mass ratio (w/b): 0.46, 0.50, and 0.54. Details of mix proportions are given in Table 3.

For group Ⅱ, based on the range analysis of the fluidity and the compressive strength (3 and 28 d) acquired by orthogonal experiment, mortars with optimal mixing ratios were selected and tested (Table 4).

For group Ⅲ, experiments were designed to analyze the influence of calcium compounds (CaO, CaSO_4_, and CaCl_2_) with different anionic species on mortar, in which water to solids (SR mixed with GGBFS, and one or more of CaO, CaSO_4_, and CaCl_2_) mass ratio is 0.5, as shown in Table 5.

In preparation for the experiments, the raw SR was first crushed and dried in an oven at 105 °C for 12 h and then ground to a particle size less than 0.075 mm, with a BET surface area of 409 m^2^/kg, as described in the previous study [11]. According to GB/T 17671-1999 (ISO) standard (China) [38], SR, GGBFS, and admixture powders were firstly well mixed for 3 min, and then water was added and mixed for another 3 min. After that, the ISO standard sand was added and mixed for 3 min to prepare fresh homogenized mortars. The mortars were molded into 40 mm × 40 mm × 160 mm prismatic molds with two layers. Each layer was vibrated for 1 min to remove entrained air. The prepared mortars with molds were then placed in sealed plastic bags to avoid the loss of water and prevent carbonization and cured in a chamber at 25 ± 3 °C and 95 ± 5% RH (relative humidity). After 24 h curing, the mortars were demolded and further cured until testing.

### 2.3. Testing and Characterization

The fluidity of designed fresh mortars was measured by a fluidity tester (NLD-3, Cangzhou Jingruida Test Instrument Co. Ltd., Cangzhou, China) as illustrated in the literature [41] and the Chinese standard GB/T 2419-2005 [42]. According to the GB/T 17671-1999 (ISO) standard (China) [43] and the literature [12], the compressive and flexural strength of specimens were measured by a model YAW-300 machine (Shanghai Suns Machinery Manufacturing Co., Ltd., Shanghai, China) with 2400 and 50 N/s loading rate, respectively. The porosity of mortars was measured according to the ASTM C20-2000 (2005) standard [44] and the literature [13]. Fluidity, porosity, compressive strength, and flexural strength were measured and averaged from 3, 3, 6, and 3 specimens, respectively. The XRD patterns were recorded on a Rigaku D/Max-2500 X-ray diffractometer (Akishima, Tokyo, Japan) with CuKα radiation using a generator voltage of 40 kV and 150 mA, from 6° to 40° at 4°/min. The microstructure of mortars was tested by SEM (Quanta JEOL JSM-7610FPlus, Rigaku, Tokyo, Japan). SEM images were taken at 2 kV, 10,000×.

## 3. Results and Discussion

### 3.1. Orthogonal Experiment

#### 3.1.1. Fluidity

**Figure 2:**
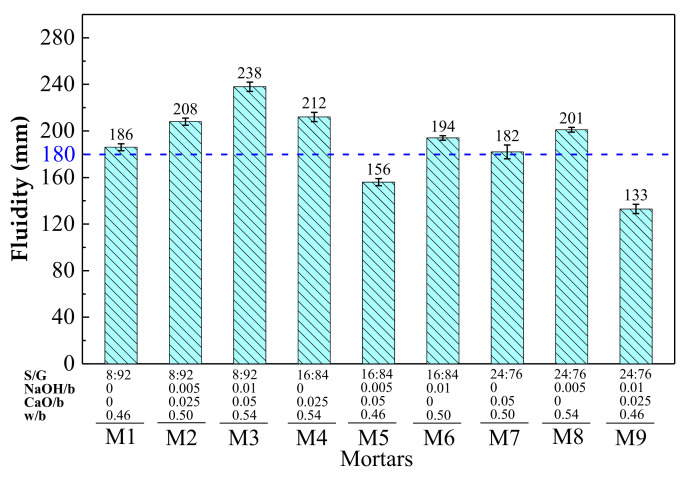
Fluidity of flesh mortars by orthogonal experiment.

The fluidity of freshly mixed mortar determines whether the mortar can be mixed, placed, consolidated, and finished. According to the GB 175-2007 standard (China) [45], the fluidity of fresh mortar should be no less than 180 mm. The fluidity of fresh mortars by orthogonal experiment is presented in Figure 2, where the fluidity of mortars varies greatly with different S/G, NaOH/b, CaO/b and w/b.

The range analysis results of fluidity by orthogonal experiment are shown in Table 6. The ranking sequence of the factors influencing fluidity is w/b > S/G > CaO/b > NaOH/b. The range results (R_j_) of w/b and S/G are much higher than those of CaO/b and NaOH/b, indicating that w/b and S/G play a major role in the fluidity of mortar.

With the increase of w/b, the fluidity of mortar increases. It is because that with the increase of free water, SR and GGBFS particles are better dispersed in water, reducing the probability of physical agglutination in mortar. It is worth noting that the fluidity of most mortars meets the standard except that of M5 and M9, with a low w/b (0.46), as shown in Figure 2.

With the increase of S/G, the fluidity of mortar decreases. Study [24] found that the physical water absorption of SR was the key factor causing the decrease of mortar fluidity. With the increase of S/G, more free water is entrapped in the pores of SR, which is adverse to fluidity. Thus, when SR proportion is increased, w/b should also be increased to ensure good workability of mortar.

#### 3.1.2. Mechanical Strength

**Figure 3:**
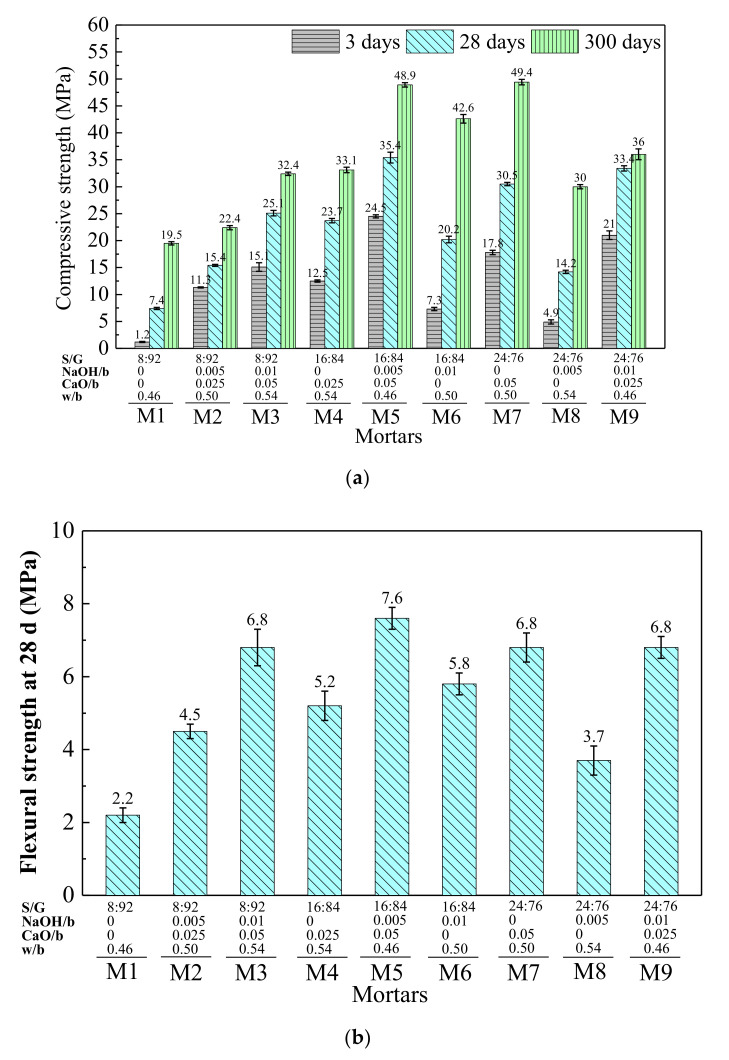
Orthogonal experiment results of (**a**) compressive strength at 3, 28, and 300 d; (**b**) flexural strength at 28 d.

The results of mechanical properties by orthogonal experiment (compressive strength at 3, 28, and 300 d, and flexural strength at 28 d) are shown in Figure 3. It can be found that the compressive strength of all mortars increases with the increase of curing age and is much higher at 300 d. Among all the mortars, M5 shows the highest compressive strength at 3 and 28 d (24.5 and 35.4 MPa) and flexural strength at 28 d (7.6 MPa); M7 shows the highest compressive strength at 300 d. When S/G is 8:92, the early compressive strength (3 d) of M2 (11.3 MPa) and M3 (15.1 MPa) is much higher than that of M1 (1.2 MPa). It indicates that the increase of NaOH, CaO, and water improves the compressive strength of SR-activated GGBFS mortar.

The range analysis results of mechanical properties by orthogonal experiment are shown in Table 7.

Ranking sequences of factors influencing compressive strength of the mortars at 3, 28, and 300 d are CaO/b > S/G > w/b > NaOH/b, CaO/b > S/G > NaOH/b > w/b, and S/G > CaO/b > w/b > NaOH/b, respectively. It can be seen that S/G and CaO/b have a greater influence on compressive strength during the whole curing time, whereas w/b and NaOH/b have a lesser influence. With the increase of CaO/b, compressive strength at 3, 28, and 300 d increases and reaches its maximum value when CaO/b is 0.05. With the increase of S/G, compressive strength (3, 28 and 300 d) increases first and then decreases. When S/G is 16:84, compressive strength reaches its maximum value. The mean values of levels 2 and 3 at 3, 28, and 300 days were 14.767 MPa and 14.567 MPa, 26.433 MPa and 26.033 MPa, 41.533 MPa and 38.467 MPa, respectively. It can be found that the differences in mean values between levels 2 and 3 in each age were almost negligible. Therefore, it can be concluded that when S/G is in the range of 16:84–24:76, the mortars activated by SR can obtain higher compressive strength. With the increase of w/b, compressive strength at 3 d and 28 d decreases, while compressive strength at 300 d increases first and then decreases and reaches its maximum value when w/b is 0.50. Considering the long-term compressive strength and workability of fresh mortars, the optimal value of w/b is 0.50. NaOH/b has little effect on compressive strength, and the maximum value of compressive strength is reached when NaOH/b is 0.01 at each age. Therefore, for compressive strength, the optimal scheme is 16:84–24:76 S/G, 0.01 NaOH/b, 0.05 CaO/b, and 0.50 w/b.

The ranking sequence of factors influencing flexural strength of the mortars at 28 d is CaO/b > NaOH/b > S/G> w/b. CaO/b and NaOH/b have a greater effect on flexural strength at 28 d, and the optimal value of CaO/b is 0.05 and 0.01. When S/G is 16:84, flexural strength reaches its maximum value. When S/G is 24:76, a slight strength decrease was found. Flexural strength at 28 d reaches its maximum value when w/b is 0.50. Therefore, for flexural strength, the optimal scheme is 16:84–24:76 S/G, 0.01 NaOH/b, 0.05 CaO/b, and 0.50 w/b at 28 d.

In conclusion, for mechanical properties, the optimal scheme is 16:84–24:76 S/G, 0.01 NaOH/b, 0.05 CaO/b, and 0.50 w/b.

### 3.2. Optimized Experiment of Mortars

According to the test results and the analysis in Section 3.1, it can be known that when S/G is 16:84 or 24:76, NaOH/b is 0.01, CaO/b is 0.05, and w/b is 0.50, the mortars can obtain high compressive strength. Thus, the optimal mortars, O1 (S/G, NaOH/b, CaO/b, and w/b are 16:84, 0.01, 0.05, and 0.50, respectively), O2(S/G, NaOH/b, CaO/b, and w/b are 24:76, 0.01, 0.05, and 0.50, respectively) are chosen to do further tests. Compared with O1, the SR in O2 is increased, which will lead to a decrease in the fluidity of the mortar. In order to increase the amount of solid waste SR in the mortar and ensure the fluidity of the mortar, it is necessary to design another optimal mortar, O3 (S/G, NaOH/b, CaO/b, and w/b are 24:76, 0.01, 0.05, and 0.54, respectively). The detailed test scheme is shown in Table 4, and the performance index of the mortars is shown in Table 8.

It can be seen that the fluidity of the three optimal mortars (O1, O2, and O3) is larger than 180 mm, which meets the requirements of the standard [45]. Compared with O1, the compressive strength of O2 is higher, indicating that under the synergistic effect of NaOH and CaO, the increase of SR is beneficial to the improvement of compressive strength. It may be because SR has strong water absorption, and the increase of SR decreases the w/b of mortar. According to the orthogonal experiment, the decrease of w/b is conducive to the improvement of compressive strength. Similarly, the reason why the compressive strength of O3 decreases, compared with O2, is the increase of w/b. The increase of SR is not conducive to the improvement of flexural strength, which is because SR, with abundant pore structure, increases the porosity of mortar. Therefore, when S/G, NaOH/b, CaO/b, and w/b are 16:84–24:76, 0.01, 0.05, and 0.50, respectively, the fluidity and the compressive strength at 28 d are 181–195 mm and 32.3–35.4 MPa, respectively.

Compared with O2, M7 in an orthogonal experiment has similar values in fluidity and porosity. Additionally, though M7 also has a higher compressive strength at 28 d, it is 4.9 MPa lower than O2. M7 is likely to be more popular with the engineering community because it does not use NaOH in its raw materials, reducing the cost of mortar and the potential for alkali toxicity. Thus, the compressive strength of M7 can be improved by admixtures, which will be further explained in Section 3.3.

### 3.3. Effects of Anionic Calcium Compounds on Compressive Strength

**Figure 4:**
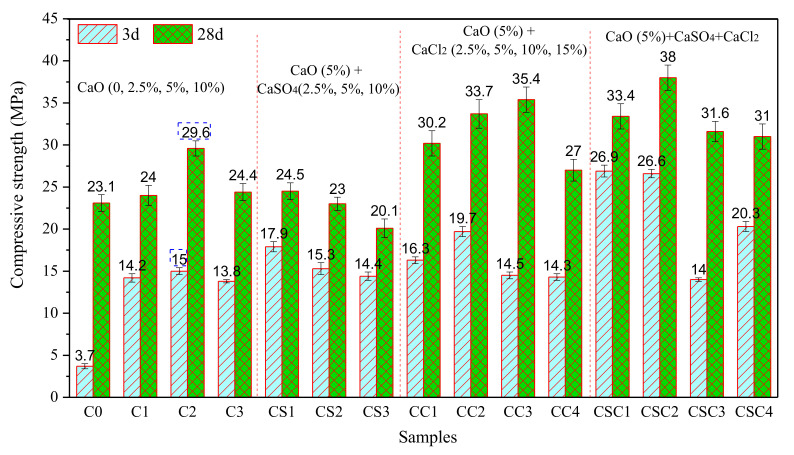
Compressive strength of SR-activated GGBFS mortars with different activators.

The orthogonal experiment results and the analysis show that CaO is beneficial to the improvement of the compressive strength of SR-activated GGBFS mortars. When CaO (5%) is added into SR (24%)-activated GGBFS (76%) mortar, the compressive strength of mortar (M7) can reach 30.5 MPa at 28 d. In order to further study the effect of different anionic calcium compounds (CaO, CaSO_4_, CaCl_2_) on the mechanical properties of SR-activated GGBFS mortars, experimental schemes were designed, as shown in Table 5. The compressive strength (28 d) of SR-activated GGBFS mortars with different activators is shown in Figure 4.

#### 3.3.1. Influence of CaO on Compressive Strength

The compressive strength of SR (24%)-activated GGBFS (76%) mortar (C0) at 3 d and 28 d is 3.7 and 23.1 MPa, respectively. The compressive strength of C0 at the early age (3 d) is very low. The addition of CaO (2.5–10%) can improve the compressive strength of mortar, especially at an early age. When the CaO proportion is 5%, the compressive strength of mortar (C2) reaches its maximum at 3 d and 28 d, 15.0 MPa and 29.6 MPa, respectively, which are about 4 times and 1.3 times higher than those of C0. Relevant studies [36,37] show that CaO can accelerate the hydration of GGBFS. CaO reacts with water to generate Ca(OH)_2_, which consumes water in the solution, further enhances local alkalinity, and accelerates the release of calcium, aluminum, and silicon phases from GGBFS particles, resulting in the generation of hydration products, such as CSH and CAH gel. It is worth noting that when the proportion of CaO is 10%, the compressive strength of mortar (C3) is improved compared with that without CaO (C0). However, compared with C2, where the CaO proportion is 5%, the compressive strength of C3 at 3 and 28 d is significantly reduced. It may be because excessive CaO cannot fully disperse in mortar, resulting in the agglomeration of CaO, which becomes the weak link of mortar, and leads to the decline of compressive strength [46].

#### 3.3.2. Influence of CaSO_4_ on Compressive Strength

On the basis of C2, the effect of CaCl_2_ on the compressive strength of CaO-SR-activated GGBFS mortars (CC1, CC2, CC3, CC4) was discussed by adding CaCl_2_ (2.5%, 5%, 10%, and 15%, respectively). It can be seen that a low proportion of CaSO_4_ (2.5–5%) is beneficial to the improvement of compressive strength of mortar at early age, whereas a 10% addition of CaSO_4_ decreases the compressive strength at an early age. The compressive strength of mortars (CS1, CS2, and CS3) with CaSO_4_ (2.5%, 5%, and 10%) at 28 d is lower than that of C2. It may be because with the increasing addition of CaSO_4_, [SO_4_]^2−^ in mortars increases. Under the alkaline environment, Ca^2+^ and [AlO_4_]^5−^ are released from GGBFS rapidly and react with [SO_4_]^2−^ to produce a large amount of ettringite (Equation (3)), which inserts into the pores of mortar and serves as a powerful skeleton structure, and improves early compressive strength. However, in the progress of the reaction, a large amount of ettringite is attached to the surface of SR and GGBFS. Further hydration of GGBFS is prevented, resulting in weaker compressive strength at 28 d. Additionally, Zhang [39] believes that when CaSO_4_ in alkali-activated GGBFS exceeds 2%, a large amount of ettringite will generate. The expansion of ettringite will lead to the concentration of stress in mortar, and the decrease of volume stability, thus reducing the compressive strength of mortar. According to the mineral composition of SR in Section 2.1 and the proportion of SR in C2, by calculation, C2 contains 2.35% CaSO_4_, which exceeds the requirement of CaSO_4_ addition (2%) in a previous study [39]. It can be seen that when SR addition is 24%, extra addition of CaSO_4_ is harmful to the development of the compressive strength of mortar.

3CaO + Al_2_O_3_ + 3CaSO_4_ + 32H_2_O → 3CaO·Al_2_O_3_·3CaSO_4_·32H_2_O (3)

#### 3.3.3. Influence of CaCl_2_ on Compressive Strength

On the basis of C2, the effect of CaCl_2_ on the compressive strength of CaO-SR-activated GGBFS mortars (CC1, CC2, CC3, CC4) was discussed by adding CaCl_2_ (2.5%, 5%,10%, and 15%, respectively). It can be seen that 2.5–5% addition of CaCl_2_ is beneficial to the improvement of the early compressive strength of the CaO-SR-activated GGBFS mortar. It is because CaCl_2_ can accelerate the generation of CSH gel and Fs, which is conductive to compressive strength [20]. When the proportion of CaCl_2_ exceeds 5%, the early compressive strength decreases, which may be because excessive CaCl_2_ leads to the existence of a large amount of Ca^2+^ ions in the solution. Under the common ion effect [47], the pH value of the solution reduces, and calcium, aluminum, and silicon phases dissolved from the GGBFS are not enough to generate CSH and Fs. The compressive strength of the CaO-SR-activated GGBFS mortar at 28 d increases first and then decreases with the addition of CaCl_2_ from 2.5% to 15%, and reaches its maximum value of 35.4 MPa when the addition of CaCl_2_ is 10%. When CaCl_2_ addition is 15%, the 28 d compressive strength is only 27 MPa, lower than that of C2 (29.6 MPa). Therefore, a 2.5–10% CaCl_2_ addition is beneficial to the improvement of compressive strength.

#### 3.3.4. Influence of CaSO_4_ and CaCl_2_ on Compressive Strength

On the basis of C2, an experiment was carried out to study the influence of composite activator, including CaSO_4_ and CaCl_2_, on compressive strength of mortars (CSC1, with 2.5% CaSO_4_ and 2.5% CaCl_2_; CSC2, with 2.5% CaSO_4_ and 5% CaCl_2_; CSC3, with 5% CaSO_4_ and 5% CaCl_2_; CSC4, with 2.5% CaSO_4_ and 10% CaCl_2_). When the addition of CaSO_4_ is 2.5%, the compressive strength of the mortars increases first and then decreases with the addition of CaCl_2_, rising from 2.5% to 10%. When CaCl_2_ addition is 5% (CSC2), the maximum value of compressive strength at 28 d is 38.0 MPa. Compared with CSC2, CaSO_4_ in CSC3 increases to 5%, and the compressive strength of the mortar at each age (3 d and 28 d) decreases significantly. It can be seen that a low proportion of CaSO_4_ and CaCl_2_ are conducive to the compressive strength of CaO-SR-activated GGBFS mortars.

### 3.4. Hydration Products

#### 3.4.1. XRD Analysis

**Figure 5:**
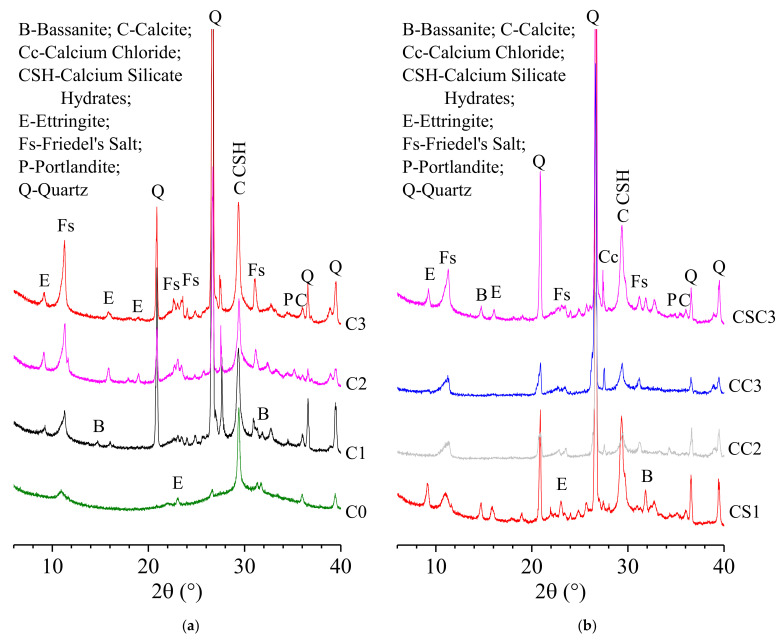
XRD patterns of (**a**) C0, C1, C2, and C3 at 28 d; (**b**) CS1, CC2, CC3, and CSC3 at 28 d.

The XRD patterns of C0, C1, C2, C3, CS1, CC2, CC3, and CSC3 at 28 d are shown in Figure 5. It can be seen from Figure 5a that the main mineral phases in C0 (SR (24%)-activated GGBFS mortar) are bassanite, calcium chloride, CSH gel, ettringite, Fs, portlandite and quartz. CSH gel, ettringite, Fs, and portlandite are hydration products of C0, which is consistent with the previous research [13]. The mineral phases, bassanite and calcium chloride come from SR, and quartz comes from ISO standard sand. Compared with C0, the XRD pattern of C1 shows a new characteristic reflection of ettringite (2θ = 9.249°, d = 0.96 nm), indicating that 2.5% CaO addition accelerates the hydration of GGBFS, and more Ca^2+^ and [AlO_4_]^5−^ released from GGBFS react with [SO_4_]^2−^ to produce ettringite. In terms of compressive strength, C1 is stronger than C0. In XRD patterns of C2 and C3, the characteristic reflections of bassanite disappear, indicating that the bassanite in SR is completely consumed to generate ettringite.

Compared with C2, in the XRD pattern of CS1, with 2.5% CaSO_4_ addition, the characteristic reflections of bassanite (2θ = 14.739°, d = 0. 06 nm; 2θ = 31.811°, d = 0.28 nm) appear, indicating that CaSO_4_ in mortar is excessive. Compared with C2, in the XRD patterns of CC2 and CC3, with 5% and 10% CaCl_2_ addition, fewer ettringite reflections are found. It may be due to more Ca^2+^ and [AlO_4_]^5−^ being released from GGBFS, and ettringite converts into Fs in the presence of high concentrations of CaCl_2_ [48], as shown in Equations (4) and (5) [47,49]. However, when CaSO_4_ and CaCl_2_ with equal mass are added, both ettringite and Fs exist in the hydration of CSC3. It can be seen that a high concentration of CaCl_2_ can reduce the amount of ettringite in mortar.

3CaO·Al_2_O_3_·3CaSO_4_·32H_2_O + 6CaO + 2Al_2_O_3_ + 4H_2_O → 3(3CaO·Al_2_O_3_·CaSO_4_·12H_2_O) (4)

3CaO·Al_2_O_3_·CaSO_4_·12H_2_O + 2Cl^−^ → 3CaO·Al_2_O_3_·CaCl_2_·10H_2_O + [SO_4_]^2−^ + 2H_2_O (5)

#### 3.4.2. SEM Analysis

**Figure 6:**
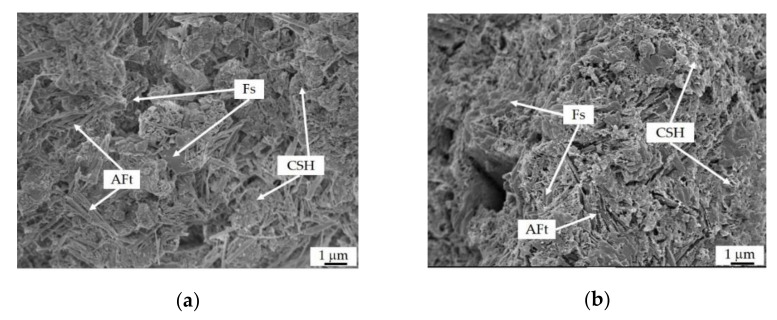

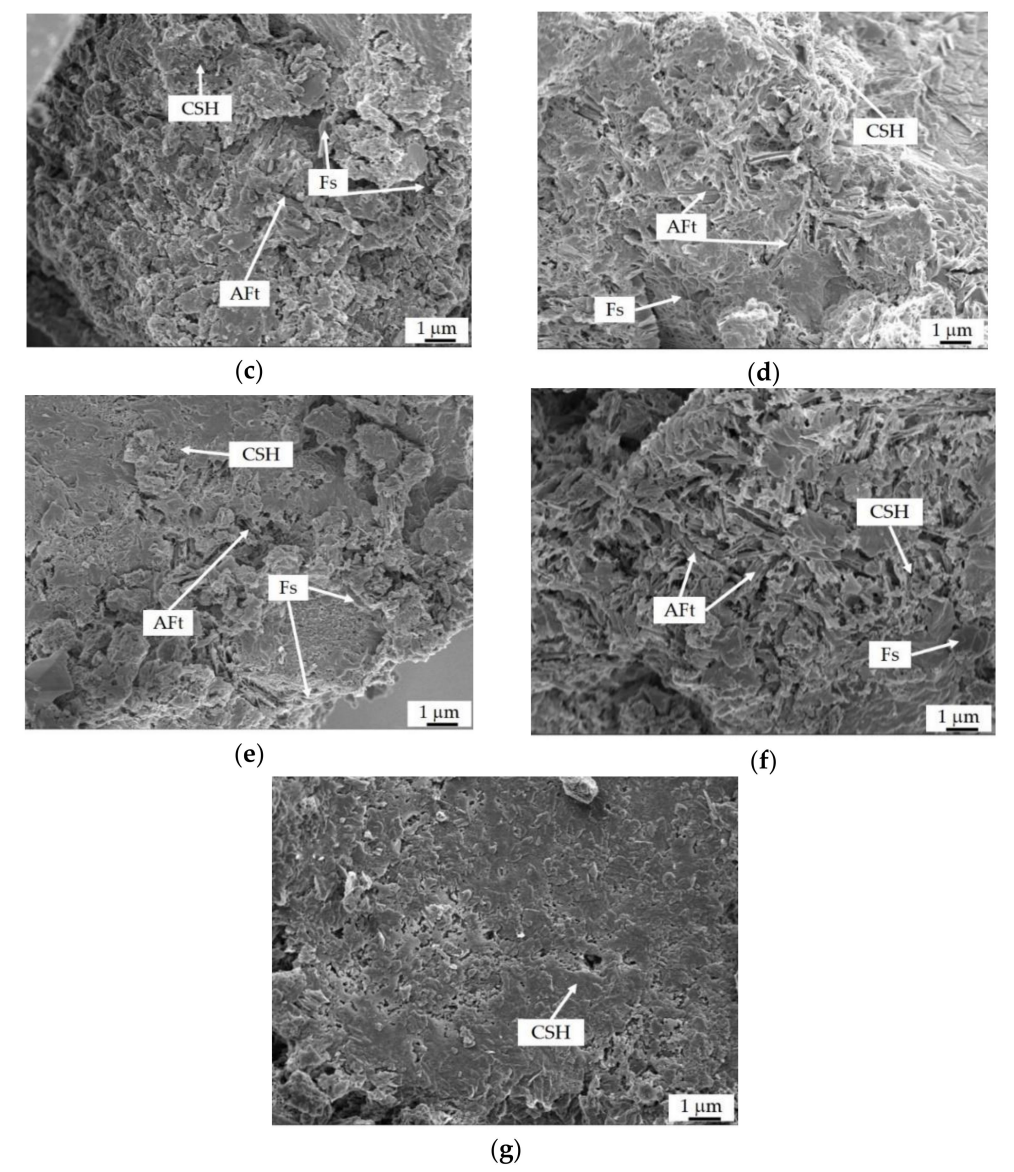
SEM images of mortars at 28 d: (**a**) C0; (**b**) C1; (**c**) C2; (**d**) C3; (**e**) CS1; (**f**) CC2; (**g**) CSC3.

Figure 6 shows SEM images of mortars (C0, C1, C2, C3, CS1, CC2, and CSC3) with different activators at 28 d. The microstructure of C0 (Figure 6a) has a large amount of needle-like ettringite (AFt), flat hexagonal Fs, and honeycomb-like CSH gels, filling the pores or adhering to the particles of SR, GGBFS, and sand. However, a large number of pores still exist, and the microstructure of C0 is not dense. With the increase of the addition of CaO from 2.5% to 10%, AFt, Fs, and CSH gels increase and fill the pores among the particles of mortars (C1–C3, Figure 6b–d). Additionally, AFt and Fs are covered with the CSH gels. The addition of 2.5% CaSO_4_ to mortar (Figure 6e) can also improve the hydration of GGBFS and generate more AFt and CSH gels. With the addition of 5% CaCl_2_ to mortar (Figure 6f), more Fs and CSH gels generate, but less Aft. In CSC3 (Figure 6g), under the influence of CaSO_4_ and CaCl_2_, the microstructure of the mortar is denser, macroscopically exhibiting higher compressive strength (38.0 MPa) at 28 d, which is in consistence with the results described in Section 3.3.4.

## 4. Conclusions

In this study, three groups of experiments are designed, including an orthogonal experiment with 4 factors and 3 levels, an optimized experiment, and an experiment on SR-activated GGBFS mortars with different anionic calcium compounds activators. Fluidity, mechanical strength, porosity, hydration products, and microstructure of mortars were studied. Based on the obtained results, the following conclusions are made.

1. The ranking sequences of factors influencing compressive strength of the mortars at 3, 28, and 300 d are CaO/b > S/G > w/b > NaOH/b, CaO/b > S/G > NaOH/b > w/b, and S/G > CaO/b > w/b > NaOH/b, respectively;
2. S/G and CaO/b have a greater influence on compressive strength during the whole curing time. When CaO/b is 0.05 and S/G is 16:84–24:76, the mortars activated by SR can obtain high compressive strength;
3. For mechanical properties and fluidity, the optimal scheme of SR-activated GGBFS mortars is 16:84–24:76 S/G, 0.01 NaOH/b, 0.05 CaO/b, and 0.50 w/b. Compressive strength at 28 d and fluidity of the mortars are 32.3–35.4 MPa, and 181–195 mm, respectively;
4. Compressive strength of CaO (5%)-SR (24%)-activated GGBFS mortars (C2) at 3 d and 28 d can reach 15.0 MPa and 29.6 MPa, respectively. Between 2.5–10% CaCl_2_ addition is beneficial to the improvement of compressive strength, whereas CaSO_4_ is harmful;
5. The main hydration products of mortars are AFt, Fs, and CSH gels. High concentration of CaCl_2_ can reduce the amount of ettringite in mortars.

## Figures and Tables

**Table 1:** Purity of admixture.

Reagents	Purity (%)	Solubility	Production Place
NaOH	96.0	soluble	Tianjin, China
CaO	98.0	slightly soluble	Tianjin, China
CaCl_2_	96.0	soluble	Tianjin, China
CaSO_4_	97.0	slightly soluble	Tianjin, China

**Table 2:** Group Ι, 4 factors and 3 levels selected for the experimental design.

Factor	Level l	Level 2	Level 3
S/G	8:92	16:84	24:76
NaOH/b	0	0.005	0.01
CaO/b	0	0.025	0.05
w/b	0.46	0.50	0.54

**Table 3:** Group Ι, mixing proportions of mortars according to the experimental design in Table 2 (g).

No.	SR	GGBFS	NaOH	CaO	Water	Sand
M1	36	414	0	0	207	1350
M2	36	414	2.25	11.25	225	1350
M3	36	414	4.50	22.50	243	1350
M4	72	378	0	11.25	243	1350
M5	72	378	2.25	22.50	207	1350
M6	72	378	4.50	0	225	1350
M7	108	342	0	22.50	225	1350
M8	108	342	2.25	0	243	1350
M9	108	342	4.50	11.25	207	1350

**Table 4:** Group Ⅱ, optimal experiment of mortars (g).

No.	SR	GGBFS	NaOH	CaO	Water	Sand
O1	72	378	4.50	22.50	225	1350
O2	108	342	4.50	22.50	225	1350
O3	108	342	4.50	11.25	243	1350

**Table 5:** Group Ⅲ, mixing proportions of SR-activated GGBFS mortars (g).

No.	SR	GGBFS	CaO	CaSO_4_	CaCl_2_	Water	Sand
C0	108	342	-	-	-	225.0	1350
C1	108	342	11.25	-	-	230.6	1350
C2	108	342	22.50	-	-	236.3	1350
C3	108	342	45.00	-	-	247.5	1350
CS1	108	342	22.50	11.25	-	241.9	1350
CS2	108	342	22.50	22.50	-	247.5	1350
CS3	108	342	22.50	45.00	-	258.8	1350
CC1	108	342	22.50	-	11.25	241.9	1350
CC2	108	342	22.50	-	22.50	247.5	1350
CC3	108	342	22.50	-	45.00	258.8	1350
CC4	108	342	22.50	-	67.50	270.0	1350
CSC1	108	342	22.50	11.25	11.25	247.5	1350
CSC2	108	342	22.50	11.25	22.50	253.1	1350
CSC3	108	342	22.50	22.50	22.50	258.8	1350
CSC4	108	342	22.50	11.25	45.00	264.4	1350

**Table 6:** Orthogonal extreme difference analysis of fluidity (mm).

Index	Factors				Rank
	S/G	NaOH/b	CaO/b	w/b	
M_1j_	211	193	194	158	w/b > S/G > CaO/b > NaOH/b
M_2j_	187	188	184	195	
M_3j_	172	188	192	217	
R_j_	39	5	9	59	
Trend	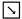	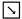 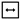	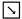 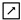	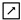	

Note: M_ij_ is average value of measured data of orthogonal experiment at i level and j column; R_j_ = M_jmax_ − M_jmin._ ‘
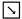
’, ‘
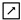
’, and ‘
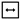
’ represent the increasing trend, the decreasing trend, unchanged, respectively.

**Table 7:** Orthogonal extreme difference analysis of mechanical strength (MPa).

Index	Index	Factors				Rank
		S/G	NaOH/b	CaO/b	w/b	
3 d Compressive Strength	M_1j_	9.200	10.500	4.467	15.567	CaO/b > S/G > w/b > NaOH/b
	M_2j_	14.767	13.567	14.933	12.133	
	M_3j_	14.567	14.467	19.133	10.833	
	R_j_	5.567	3.967	14.666	4.734	
	Trend	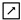 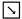	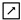	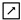	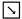	
28 d Compressive Strength	M_1j_	15.967	20.533	13.933	25.400	CaO/b > S/G > NaOH/b > w/b
	M_2j_	26.433	21.667	24.167	22.033	
	M_3j_	26.033	26.233	30.333	21.000	
	R_j_	10.466	5.700	16.400	4.400	
	Trend	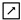 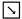	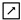	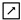	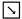	
300 d Compressive Strength	M_1j_	24.767	34.000	30.700	34.800	S/G > CaO/b > w/b > NaOH/b
	M_2j_	41.533	33.767	30.500	38.133	
	M_3j_	38.467	37.000	43.567	31.833	
	R_j_	16.766	3.233	13.067	6.300	
	Trend	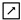 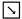	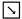 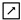	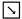 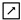	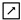 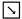	
28 d Flexural Strength	M_1j_	4.500	4.733	3.900	5.533	CaO/b > NaOH/b > S/G > w/b
	M_2j_	6.200	5.267	5.500	5.700	
	M_3j_	5.767	6.467	7.067	5.233	
	R_j_	1.700	1.734	3.167	0.467	
	Trend	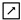 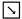	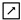	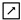	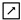 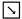	

**Table 8:** Performance index of mortars.

No.	Fluidity (mm)	Porosity (%)	Compressive Strength (MPa)		28 d Flexural Strength (MPa)
			3 d	28 d	
O1	191 ± 4	20.5 ± 0.5	22.9 ± 0.6	32.3 ± 0.4	8.0 ± 0.2
O2	181 ± 3	21.2 ± 0.4	24.3 ± 1.1	35.4 ± 0.9	7.1 ± 0.3
O3	195 ± 4	22.7 ± 0.6	21.9 ± 0.8	33.0 ± 0.7	7.1 ± 0.4
M7	182 ± 6	21.9 ± 0.5	17.8 ± 0.4	30.5 ± 0.3	6.8 ± 0.4

## Data Availability

Not applicable.
